# Does a free maternity policy in Kenya work? Impact and cost–benefit consideration based on demographic health survey data

**DOI:** 10.1007/s10198-023-01575-w

**Published:** 2023-02-13

**Authors:** Boniface Oyugi, Olena Nizalova, Sally Kendall, Stephen Peckham

**Affiliations:** 1 Centre for Health Services Studies (CHSS), University of Kent, George Allen Wing, Canterbury, CT2 7NF England; 2 University of Nairobi, College of Health Sciences, P.O BOX 19676-00202, Nairobi, Kenya; 3 Personal Social Services Research Unit (PSSRU), University of Kent, Cornwallis Central, Canterbury, CT2 7NF England; 4 School of Economics, University of Kent, Kennedy Building, Canterbury, CT2 7FS England

**Keywords:** Free maternity policy, Perinatal mortality, Neonatal mortality, C01, I10, I18

## Abstract

This paper evaluates the overall effect of the Kenyan free maternity policy (FMP) on the main outcomes (early neonatal and neonatal deaths) and intermediate outcomes (delivery through Caesarean Section (CS), skilled birth attendance (SBA), birth in a public hospital and low birth weight (LBW)) using the 2014 Demographic Health Survey. We applied the difference-in-difference (DID) approach to compare births (to the same mothers) happening before and after the start of the policy (June 2013) and a limited cost–benefit analysis (CBA) to assess the net social benefit of the FMP. The probabilities of birth resulting in early neonatal and neonatal mortality are significantly reduced by 17–21% and 19–20%, respectively, after the FMP introduction. The probability of birth happening through CS reduced by 1.7% after implementing the FMP, while that of LBW birth is increased by 3.7% though not statistically significant. SBA and birth in a public facility did not moderate the policy’s effects on early neonatal mortality, neonatal mortality, and delivery through CS. They were not significant determinants of the policy effects on the outcomes. There is a significant causal impact of the FMP in reducing the probability of early neonatal and neonatal mortality, but not the delivery through CS. The FMP cost-to-benefit ratio was 21.22, and there were on average 4015 fewer neonatal deaths in 2013/2014 due to the FMP. The net benefits are higher than the costs; thus, there is a need to expand and sustainably fund the FMP to avert more neonatal deaths potentially.

**Supplementary Information:**

The online version contains supplementary material available at 10.1007/s10198-023-01575-w.

## Introduction

**Fig. 1:**
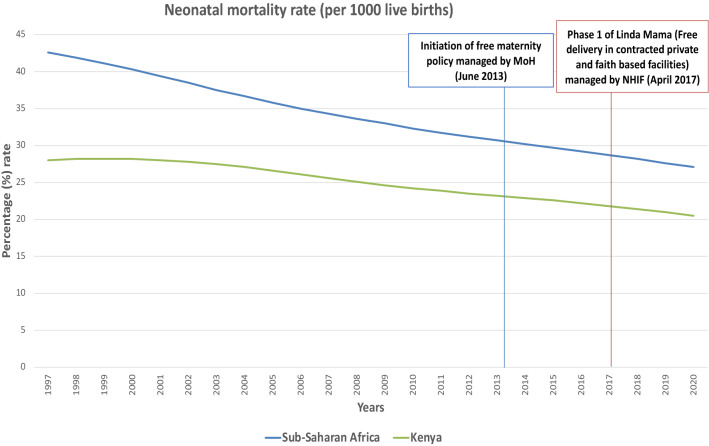
Neonatal mortality rates (per 1000 live births), 1997–2020. (Source: Prepared by authors using datasets from https://databank.worldbank.org/source/world-development-indicators# )

Despite significant progress in improving the maternal and child health status by different countries in the last decade, preventable maternal and neonatal deaths remain a global health challenge [1]. Every day, approximately 810 women die from preventable causes related to pregnancy and childbirth [2], almost 6,700 newborn deaths [3] and more than 7000 stillbirths [4]. Low-income countries (LIC) and low-and-middle-income countries (LMIC), particularly those in Sub-Saharan Africa (SSA), are the most affected because of the poor access to and utilisation of maternal and family planning services. Countries in SSA, such as Kenya, have significantly improved maternal and child health in the last decade (maternal mortality ratio has decreased by 52% from 2000 to 2017 [2], while the neonatal mortality rate has reduced from 33 to 22 deaths per 1000 live births between 1990 and 2014 [5]). Nevertheless, mothers and neonates still die from preventable pregnancy-related complications [5]. To address this issue further, Kenya has set on a path of reducing catastrophic expenditure on maternity care by individuals through investment in a free delivery (birth) policy as a step towards UHC. In June 2013, the government of Kenya (GoK) initiated a waiver of the user fees payable for all maternity and primary health care services, which aimed to reduce maternal mortality via improving access to skilled birth attendance (SBA) in public facilities [6] (Fig. 1).

Following the free maternity policy (FMP) implementation, there was an estimated 10% increase in the total number of deliveries across the country by July 2013, with increases of 50% in certain counties [7]. A process evaluation of the FMP, conducted in three purposefully selected counties between July 2015 and January 2016, showed that it was haphazardly implemented without adequate equipment and preparation of the public hospitals to meet the increased number of mothers who came for delivery due to the free policy [8]. Additionally, there were no adequate systems to verify the quality of care due to the policy and the reimbursement claims from the hospitals to the government [8]. Subsequently, in 2017, the GoK moved the management of the FMP from the Ministry of Health to the National Hospital Insurance Fund, aimed at using it as a driver towards UHC [9]. This move led to a phased approach to implementing the expanded free maternity services programme dubbed *Linda Mama (LM)* (Swahili word for taking care of the mother), aiming at providing free access to service delivery for all pregnant women in private, faith-based, and public institutions (Fig. 1).

The goal of the FMP was to provide access to free labour and delivery services to improve maternal and infant health outcomes. Some studies from Kenya on the FMP provided a descriptive analysis of the FMP effect on neonatal outcomes using cross-sectional data obtained from 77 health facilities [10–12]. Studies that relied on an interrupted time series analysis (2 years before and 2 years after the policy) produced mixed evidence. One documented no significant increase in the CS rates following the introduction of the FMP in 2013 [13], while another showed a level increase in normal deliveries and CS followed by a trend increase in CS in public facilities [14]. While these studies have attempted to elucidate the causal effect, they have limitations in achieving causal estimates of the FMP. Using the cross-sectional data from a select number of facilities in selected counties may have potentially under or overestimated the effect on maternal and neonatal outcomes. Equally, using Kenya Health Information System data in one study has a limitation: the data do not always conform to the information reported in the facility records. Besides, their methods were descriptive and only compared the differences before and after the policy implementation or a time series with data that had gaps. To address these limitations, we applied a difference-in-difference approach to individual-level nationally representative data to compare the birth outcomes of the same mother that occurred before and after the FMP implementation.

Using Kenya Demographic Health Survey (KDHS), we have evaluated the overall effect of the FMP in Kenya (implemented in 2013) on both early neonatal (the probability of dying within the first seven days of life) and neonatal mortality (the probability of dying within the first 28 days of life). We argue that neonatal mortality is the best outcome for this analysis for three reasons. First, it is a measurable outcome that can be averted by a series of quality processes and inputs within the continuum of maternal care [15, 16], which is the focus of the FMP. Second, this outcome is less prone to misreporting in the maternal history data collection process. Third, given the proximity to the actual birth, this outcome is most closely linked to the goals of the FMP. Additionally, we analyse the impact of the policy on intermediate outcomes of delivery through CS since, as noted by Yisma et al. [17], it is increasingly associated with neonatal mortality, especially for babies in poorer health; and other outcomes of SBA, birth in a public hospital and LBW. Finally, we present an exercise in cost–benefit analysis of the FMP based on the estimated effect from the analysis.

## Methods

### Study data

This study utilised the births recode file (unit of analysis is birth) of the 2014 KDHS. The 2014 KDHS data are a nationally representative survey of women of reproductive age [18, 19] collected from May 7 to October 20, 2014. The survey questionnaire asked a limited number of questions about complete fertility history and detailed questions on births within the five years preceding the data collection. We used the maternal history questions presented using variable *midx.* It contained up to 6 entries of births in the last five years before the interview, resulting in an analytical sample with information on 14,949 mothers and 20,964 births.

### The theory of change and data preparation

**Fig. 2:**
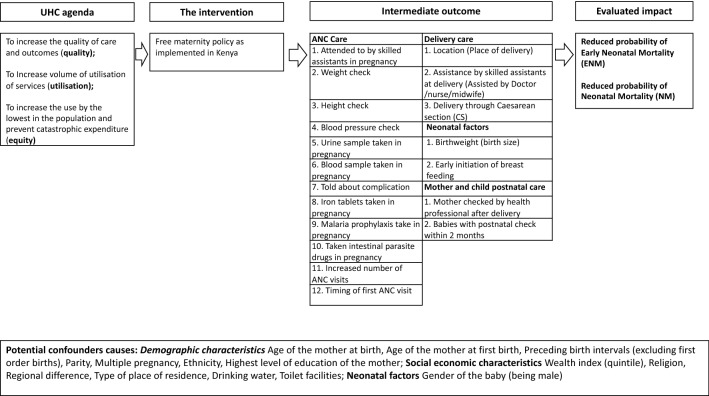
Framework for impact analysis

The theory of change underpinning this study is based on the review of the literature on different factors (ANC care, delivery care, neonatal care/factors, postnatal factors, socioeconomic, demographic, and biological) that elucidate the expected impact of the FMP on maternal and infant outcomes (see Online Appendix 1). From the literature, we curated a theory of change (Fig. 2) for this study which shows that the intervention (FMP as implemented in Kenya) seeks to achieve the UHC agenda: increasing the quality of care, the volume of utilisation of services, and prevention of catastrophic expenditure in seeking maternal care. The impact of the intervention can be adequately measured using neonatal mortality outcomes, which were chosen as the main outcomes from the policy perspective because they represent the measurable ultimate outcome that can be averted by a series of quality processes and inputs within the continuum of maternal care [15, 16]. We treat some of the determinants of neonatal mortality mentioned above as intermediate outcomes (mediators) because the policy could have affected them and, through them, affected the outcome of interest. For instance, delivery through the CS is an intermediate outcome since access to free maternity care makes it more likely for a mother to have CS, which is increasingly associated with neonatal mortality [17]. Online Appendix 2 contains a detailed narrative of the data preparation and the summary of the variables, while summary statistics are provided in Online Appendix 3.

### Empirical strategy

We used the difference-in-difference (DID) approach to estimate the impact of the FMP on two main outcomes: early neonatal and neonatal mortality that happened before and after the introduction of the FMP. Data availability restricted the choice of other intermediate outcomes –most questions were only asked about the most recent pregnancy. Therefore, this analysis was only possible for birth through CS, skilled delivery, birth in a public facility (hospital), and LBW.

The starting basic OLS regression model using data limited to children born in the last five years is denoted as:

1 $${y}_{it}={\beta }_{0}+{\beta }_{1}{after}_{it}+{\beta }_{2}{bbm}_{it}+{{\beta }_{3}{bby}_{it}+ {X}_{it}\beta }_{4}+{\varepsilon }_{it,}$$

where ${y}_{it}$ is the outcome (early neonatal death, neonatal death, and delivery by CS), which is a binary measure for newborn $i$*,* at time $t$*;* ${\beta }_{0}$ is the intercept; ${\beta }_{1}$ is the slope associated with the variable ${after}_{it}$–*birth after the policy (after* = *1);* ${bbm}_{it}$ is the birth month, and ${bby}_{it}$ is the birth year; ${X}_{it}$ is the vector of the included characteristics, and ${\varepsilon }_{it}$ is the error term. To account for potential *seasonality and cohort effects* on mortality, we added the month and the year of the births as independent variables. We use robust standard errors clustered at the individual level of the mothers.

The outcomes are associated with health inputs such as maternal characteristics, neonatal factors, and economic status from the theory of change above. Whereas utilisation of the ANC services has been shown as a significant mortality determinant in the literature review, they are effectively intermediate outcomes of the FMP because the policy essentially works through them, and they, in turn, affect the outcomes of interest. The same applies to delivery care elements, including birth location and SBA, as are neonatal factors such as LBW, early initiation of breastfeeding, and mother and child postnatal care factors. Occupation, place of residence, wealth index, region of residence, the highest level of education of the mother, and the economic status (source of drinking water and toilet availability), which are shown in the literature to determine maternal outcomes and neonatal mortality, are only measured at the time of interview. Hence, they could have been entirely different at the time of birth. Our model does not include the APGAR score as a control because it is not captured in the DHS data. Hence, the final control variables included in the models are the age of the mother at the birth of the baby, the age of the mother at the first birth, birth intervals (excluding first-order births), parity, multiple pregnancies, ethnicity, religion, and gender of the baby (male).^1^

When searching for the correct specification in the final model, we explored the joint significance of the following variables: indicator variables for birth months, ethnicity and religion using the F-test. The vector of birth months’ indicators was excluded from the regression (because the F statistic for joint significance was not greater than 0.49 in any of the specifications). The final model becomes:

2 $${y}_{it}={\beta }_{0}+{\beta }_{1}{after}_{it}+{{\beta }_{2}{bby}_{it}+ {X}_{it}\beta }_{3}+{\varepsilon }_{it}.$$

The model above represents the average treatment effect of the policy using data on all children born in the last five years. The estimates of the average treatment effect from the OLS model with all children born in the last five years have three limitations. First, as mentioned above, some of the controls mentioned in the literature are not available at the time of birth; hence the further we go in time from the interview, the more difficult it is to assume that they would not matter. Second, there could have been different things happening in Kenya that could have affected the policy (e.g. policy regimes in other areas of life, microeconomic conditions, welfare etc.); thus, the births before cannot be compared to births after for reasons unrelated to policy, and this would be exacerbated the further back we go with births included in the analysis. Third, the number of births before is much larger than the number of births afterwards; hence, technically, what happens before will dominate what happens afterwards. To address these concerns and achieve a more balanced sample, we limit the data to include only the births that happened one year before and one year after the FMP implementation.

The remaining concern is that the policy could have affected the case mix of mothers giving birth. For example, mothers with poor health endowment could have avoided giving birth before the FMP because it would have been too expensive, or they did not carry the pregnancy to birth/term. But now that it is free, mothers with poorer health endowment could have chosen to give birth, or those with at-risk pregnancies carried them to birth. All these considerations could have resulted in a completely different case mix of births after the policy compared to before, which cannot be accounted for by the observed control variables. To summarise, the pooled cross-sectional data approach (which effectively represents the simple difference before and after the policy introduction) does not allow us to account for the unobserved heterogeneity of mothers. Mothers who gave birth before could have different unobserved (to the researcher) characteristics to mothers who gave birth after, which may impact birth outcomes. If the case mix of mothers after the policy introduction has poorer health endowment, then the cross-sectional set-up would lead to an underestimation of the true effect of the policy. The greater likelihood of women with a poorer health endowment giving birth after the policy introduction will limit the magnitude of the policy effect as they are also the ones who are more likely to end up with a greater probability of a neonatal death outcome. Thus, to eliminate the bias resulting from the changing case mix, we account for the mother fixed effect (i.e. get rid of the individual mother unobserved heterogeneity) by further restricting the sample to only mothers who have at least one birth before and at least one after the FMP implementation, and, effectively, comparing pairs of births to the same mother.

Hence, we formulate the fixed effects model as follows:

3 $${y}_{it}={\beta }_{0}+{\delta }_{0}{\mathrm{after}}_{t}{+ {X}_{it}\beta }_{2}+{a}_{i}+{\varepsilon }_{it.}$$

As is in the above model, ${y}_{it}$*; *${after}_{it}$*,* and ${X}_{it}$ remain the same. ${\beta }_{0}$ is the average mortality rate before the policy introduction for *after* = *0*, and the intercept for *after* = *1* is ${\beta }_{1}+{\delta }_{0}$; and ${a}_{i}$ is the unobserved mother effect (fixed effects) and the ${\varepsilon }_{it}$ is the idiosyncratic error term. Since the observations come from the same mother, we employ robust standard errors cluster at the individual level and the fixed effects. The time-invariant mothers' characteristics are controlled for (even though it is impossible to estimate their coefficients). The estimate of the effect of the policy is derived from the comparison of the neonatal mortality outcomes (and other intermediate outcomes) between babies born to the same mother (from the restricted sample) and averaged across all mothers, i.e. the average treatment effects of the policy. This approach is preferable because it removes the time-invariant unobserved effects [20] at the mother's level from the model. Fixed effects models are helpful when '(1) time-constant unobserved heterogeneity is likely to be a problem (e.g. concerning selection into the treatment), (2) one is not interested in societal group-level differences, (3) time-varying unobserved heterogeneity is unlikely to pose a problem, and (4) the direction of the causal effect is theoretically clear (i.e. if there is no reverse causality)’ [21]. However, one key limitation of the fixed effect models is that they are 'hardly a silver bullet if the causal inference is threatened by reverse causality’ [22]. In this case, reverse causality is not a problem because, in our sample, each mother and her birth outcomes do not affect the introduction of the policy. Moreover, we are estimating within-mother effects.

Despite offering a convenient way of addressing the changes in the case mix of mothers, the fixed effect approach described above raises another concern. It may potentially overestimate the effect given that the sample after the policy would not include any women for whom this was their first birth, while the sample before would. To evaluate the importance of this concern, we restricted the sample further to include only mothers who have at least one birth—specifically second and onward births (excluding first births)—before the policy and at least one after the policy implementation. This was based on the distinct patterns of relationship between birth order and mortality in the neonatal period, where 'firstborn children are slightly more susceptible to the risk of dying young compared with children of second and third birth order' [23]. Whereas the same study shows that the last-born children (i.e. fourth- and higher-order births) are at the worst risk, the mothers in the sample utilised had not passed the childbearing age. Thus, one cannot ascertain with authority if this was their last born. In that regard, we did not exclude them from the sample—as the mothers may have potentially been planning to have more babies in the future. In this way, we are generating an experiment by making the births happening before and after more comparable because they occur to the same mothers.

To test whether the coefficients from the two methodological approaches described above, i.e. fixed effects (with firstborn sample) and fixed effects (without firstborn sample), are statistically different from each other, we employed the *z-score test* described as:

4 $$z - score = \left( {\widehat{{\delta _{0} }} - \check\delta _{0} } \right)/\surd \left( {\varepsilon _{{\widehat{{\delta _{0} }}}}^{2} + \varepsilon _{\check{\delta _{0} }}^{2} } \right),$$

where $\widehat{{\delta_{0} }}$ and $\check{{\delta _{0} }}$refer to the coefficients of *birth after the policy (after* = *1)* in the two groups of regressions (fixed effects (with firstborn sample) and fixed effects (without firstborn sample)); and $\varepsilon_{{\widehat{{\delta_{0} }}}}^{2}$ and $\varepsilon_{{\widehat{{\delta_{0} }}}}^{2}$are the corresponding standard errors. Using Eq. (4) and the estimates obtained from Eq. (3) for both the fixed effects (with the firstborn sample) and fixed effects (without the firstborn sample), we first calculated the z scores manually. Then using the *Z score and probability converter* obtained from Calculator.net [24], we interpreted the results using p(x > Z); where any *p-value* (the probability) that is greater than the critical value (0.05) means that there is no statistical difference in the estimates of the outcomes.

At this point, the design could not allow for exploration of the other intermediate outcomes (except skilled delivery, birth in a public facility, and LBW) as the corresponding DHS questions were based on the respondent’s last birth rather than births in the five years [25]. Birth in a public facility is of interest because the FMP, as implemented in 2013, was operationalised to cater for deliveries in all public facilities (including hospitals) and not private for-profit or not-for-profit facilities [6].

We applied a placebo analysis to test for the robustness of the findings to the assumptions made in the analysis [26]. First, the treatment effects in Eq. (3) apply to the same outcomes (early neonatal and neonatal mortality; and the additional intermediate outcome of delivery through CS) but applied in the unrelated (placebo or ‘fake’) time rather than when the policy took effect. We used three times that happened before the actual policy dates (as placebo): August 2012 (randomly chosen), November 2012 (randomly chosen), and March 2013 (purposefully chosen to capture the election month and formation of county governments). Statistical significance of these 'fake' treatment effects on the outcomes would reflect the differential time trends rather than the true effects of the policy. The test would show that the fixed effect model above—having considered the differential time trend—shows the true effects of the policy. An alternative procedure of the placebo test would be to use a different outcome (say, a disease or a disability) that does not relate to the births and that cannot plausibly be affected by the policy. However, the procedure was not feasible because no disease or disability outcome could be linked to the same period when the analytic sample's births happened.

### Cost–benefit considerations

We designed a limited cost–benefit analysis (herein referred to as cost–benefit consideration) to assess the net social benefit of the FMP, which answers the question: ‘*is the programme worthwhile?’* [27]. The analysis values a programme’s consequences in monetary units to allow easy direct comparison of ‘programme’s incremental cost consequences with incremental consequences incommensurate units of measurements' [27]. We used the most appropriate cost-effectiveness indicators and compared the average annual per neonatal death averted by the FMP and the average annual per neonatal benefit. Denotations and formulae for the cost–benefit consideration are in *Table **4**.*

### Ethics approval

This study was part of a more extensive study [28]. Ethical approval was obtained from the University of Kent, SSPSSR Students Ethics Committee and AMREF Scientific and Ethics Review Unit in Kenya (Ref: AMREF–ESRC P537/2018).

### Patient and public involvement

There were no patients involved in this part of the analysis as it was based on KDHS data.

## Results

### Sample characteristics and descriptive analysis

The summary statistics of the sample are provided in Online Appendix 3. A summary of the characteristics' differences before and after the FMP introduction is shown in Table 1. There are no significant differences in the proportions of early neonatal and neonatal deaths before and after the policy. However, the intermediate outcomes—delivery through CS, in a public hospital, and with a skilled attendant—show a significant difference before and after the FMP. Although most of the individual characteristics do not show a statistically significant difference before and after the policy, there are two exceptions—birth interval and ethnicity dummies. This finding provides additional support for the need to control for the confounding factors in the regression framework. Although our data comes from the nationally representative survey, to test the concerns regarding potential oversampling from better performing counties (see suggestive evidence from Tama et al. [8]), we compared the differences in the geographic representation across the regions before and after the FMP introduction. Given that we found statistically significant differences for one of the regions (Online Appendix 3 and see the more extensive study [28]), we augmented the specification with corresponding controls.

**Table 1:** Difference in the characteristics before and after (*n* = 20,927)

	Before Free Maternity	After free maternity	Diff (After – Before)	se (mean)
Dependent variable				
Early neonatal mortality	0.0157	0.0171	0.0014	0.0021
Neonatal mortality	0.0234	0.0235	0.0001	0.0025
Intermediate outcome variables (Delivery care)				
Delivery through CS	0.0646	0.0772	0.0126**	0.0041
Birth in a public hospital	0.4164	0.4840	0.0676***	0.0107
Assistance by skilled assistants at delivery (Assisted by Doctor /nurse/midwife)	0.5567	0.5980	0.0414***	0.0106
Intermediate outcome variables (Neonatal care/ factors)				
Low birth weight	0.0212	0.0210	−Done0.0002	0.0024
Independent variables				
Maternal characteristics (socioeconomic, demographic, biological)				
Age of the mother at the birth of the baby				
Less than 20	0.1502	0.1427	-0.0076	0.0059
20–34	0.7248	0.7242	−0.0006	0.0071
35 years and above	0.1250	0.1331	−0.0081	0.0054
Age of the mother at first birth (adolescent)				
18 years and below	0.4535	0.4497	−0.0038	0.0081
Preceding birth intervals (excluding first-order births)				
Less than 2 years	0.2002	0.1628	−0.0374***	0.0073
Parity				
Grand multipara	0.3975	0.3874	−0.0101	0.0080
Multiple pregnancy	0.0270	0.0308	0.0039	0.0027
Ethnicity				
Kalenjin	0.1515	0.1570	0.0055	0.0059
Kamba	0.0777	0.0800	0.0023	0.0044
Kikuyu	0.1159	0.1037	−0.0122**	0.0052
Luhya	0.1200	0.1131	−0.0069	0.0053
Luo	0.1053	0.1017	−0.0037	0.0050
Somali	0.0824	0.0796	−0.0028	0.0045
Other	0.3473	0.3650	0.0176*	0.0079
Religion				
Roman Catholic	0.1840	0.1819	−0.0021	0.0063
Other Christian	0.6196	0.6126	−0.0070	0.0079
Muslim	0.1670	0.1719	0.0049	0.0061
Other	0.0278	0.0314	0.0037	0.0027
Neonatal factors				
Gender of the baby				
Male	0.5067	0.5095	0.0028	0.0082

***p < 0.01, **p < 0.05, *p < 0.1; statistical significance next to the estimates refers to the differences in the indicators before and after the policy

### Estimation results

Table 2 allows for the comparison of the estimates of the policy effect across various specifications for two outcomes–early neonatal mortality and neonatal mortality. The estimated treatment effect based on the *OLS (model 1 in columns (1) and (5))* is neither statistically significant at a 5% level of significance nor distinguishable from zero in magnitude. However, these estimates include observations from the extended period (5 years), which may mask the true effects as the number of births before the FMP dominate the number of births after the policy. Therefore, we estimated the impact of the policy on the outcomes by restricting the sample to one year before and one year after the FMP introduction (Table 2, OLS*–model 2 in columns (2) and (6))* and still found none of the outcomes to be statistically significant. Yet, this model is still subject to concerns about the sample composition before and after the FMP. Some women who may not have given birth before the policy because of the potentially poor birth outcomes and related expenses may now have decided to give birth because of the introduction of the policy. Hence, the characteristics of the mothers before the policy could be different from those after the policy. To address these concerns and determine the true impact of the FMP, we accounted for the mother fixed effects on birth outcomes by using a sample with the same mothers who gave birth both before and after the FMP introduction.

**Table 2:** Estimation of the impact of the policy on early neonatal and neonatal mortality

	Early neonatal death				Neonatal death			
	OLS (Model 1)	OLS (Model 2)	FE (with firstborn)	FE (without firstborn)	OLS (Model 1)	OLS (Model 2)	FE (with firstborn)	FE (without firstborn)
	(1)	(2)	(3)	(4)	(5)	(6)	(7)	(8)
	*n* = 16.056	*n* = 6.653	*n* = 5.052	*n* = 1.467	*n* = 16.056	*n* = 6.653	*n* = 5.052	*n* = 1.467
Born after policy	− 0.002	− 0.002	− 0.165**	− 0.206**	0.001	0.002	− 0.193***	− 0.200**
	(0.004)	(0.004)	(0.068)	(0.081)	(0.005)	(0.005)	(0.073)	(0.082)
_cons	0.008	0.008	0.181**	0.246***	0.007	0.008	0.199**	0.227**
	(0.007)	(0.008)	(0.072)	(0.091)	(0.009)	(0.011)	(0.078)	(0.097)

***p < 0.01, **p < 0.05, *p < 0.1; Clustered robust standard errors in parentheses

*OLS* ordinary least squares; *FE* fixed effects

Model 1: Using the five-year sample (complete data set)

Model 2: Using the one year before and one year after the policy sample

FE (with firstborn): is the fixed effects when firstborns are included in the sample; while FE (without firstborn): is fixed effects when the sample is restricted only to women who have second or higher-order birth before the policy

Controls: (Year of birth, age of the mother at the birth of the baby, Preceding birth intervals of less than 2 years, Grand multipara, Multiple pregnancy, male baby, age of the mother at the first birth, ethnicity, and religion) were used as controls for the models. A complete set of estimates is available upon request

The fixed-effect estimates (Table 2*, FE (with firstborn) in columns (3) and (7)* and *FE (without firstborn) in columns (4) and (8))* suggest that the FMP introduction has reduced the probability of birth resulting in an early neonatal death by 20.6% (or by 16.5% when the firstborn are included in the model). In comparison, neonatal mortality is reduced by 20.0% (or 19.3% when firstborns are included in the model). All the estimated effects of the FMP introduction are statistically significant at a 1% level. Of all the control variables, only the birth year and an indicator for multiple pregnancy significantly affect both outcomes.

As for the effects on the intermediate outcomes, two of the three—delivery through CS and LBW—are insignificant under the FE model. The probability of birth happening through CS reduces by 1.7% after the FMP introduction, and the probability of a child being LBW increases by 3.7% (Table 3,* FE with first born (column 3)).* At the same time, the estimated effects on skilled birth attendance (SBA) and birth in a public facility (hospital) are statistically significant in all models. The probabilities of birth through SBA and in a public facility (hospital) significantly increase by 17.0% and 5.8%, respectively (Table 3,* FE with first born (column 3))* due to the FMP introduction. Of all the control variables, the multiple pregnancy significantly explains the probabilities of delivery through CS in the FE models. However, none of the four intermediate outcomes is significant when the sample is restricted to women who have second- or higher-order birth before the policy (Table 3, FE without first born (column 4)).

**Table 3:** Estimation of the impact of the policy on intermediate outcomes

	Born after policy			
	OLS (model 1)	OLS (model 2)	FE (with the firstborn)	FE (without firstborn)
	(1)	(2)	(3)	(4)
Delivery through CS	*n* = 16,056	*n* = 6646	*n* = 5037	*n* = 1461
	0.010	0.010	− 0.017	− 0.021
	(0.008)	(0.008)	(0.022)	(0.030)
Skilled delivery	*n* = 16,056	*n* = 6644	*n* = 5024	*n* = 1451
	0.041**	0.040**	0.170**	0.160
	(0.017)	(0.017)	(0.085)	(0.099)
Birth in a public facility (hospital)	*n* = 16,056	*n* = 6654	*n* = 5053	*n* = 1468
	0.058***	0.058***	0.200**	0.145
	(0.017)	(0.017)	(0.083)	(0.091)
Low birth weight	*n* = 15,982	*n* = 6654	*n* = 5053	*n* = 1468
	− 0.006	− 0.006	0.037	0.044
	(0.005)	(0.005)	(0.031)	(0.038)

The specifications are the same as Table 2 fixed effects models

***p < 0.01, **p < 0.05, *p < 0.1; Clustered robust standard errors in parentheses.

OLS: ordinary least squares; FE: fixed effects

Model 1: Using the five-year sample (complete data set)

Model 2: Using the one year before and one year after the policy sample.

FE (with firstborn): is the fixed effects when first born are included in the sample; while FE (without firstborn): is fixed effects when the sample is restricted only to women who have 2nd- or higher-order birth before the policy

Controls: (Year of birth, age of the mother at the birth of the baby, Preceding birth intervals of less than 2 years, Grand multipara, Multiple pregnancy, male baby, age of the mother at the first birth, ethnicity, and religion) were used as controls for the models. A complete set of estimates is available upon request

The z-score test for the equality of the estimates produced by the two FE models (with and without firstborns) was conducted (as described above) and showed no statistically significant difference between them. Hence, in what follows, we use the fixed-effect specification with mothers who have at least one child born before and at least one child born after the policy introduction without restrictions as to the birth order as our preferred specification.

### Exploration of the model

As the analysis of the intermediate outcomes (Online Appendix 4) suggests*,* most of the effect has happened because of skilled delivery (~ 17% out of 20%), with the remainder of the effect possibly attributed to other mechanisms such as quality of care (neonatal and maternal), availability of antenatal care and identification of possible complications earlier on in pregnancy, which need to be explored in the future.

Online Appendix 5 presents the estimates from the preferred model when the FMP is defined using a placebo time cut-off point described in the methods. There is no documented statistically significant impact of the placebo treatment (the other randomly chosen implementation timings of August 2012, November 2012, and March 2013 of the FMP (placebo) introduction) on neonatal outcomes (early neonatal and neonatal mortality) and delivery through CS as intermediate outcomes.

### Cost–benefit considerations

The average annual maternity cost of the FMP was estimated at USD 43.7 million, the amount spent on the policy in the financial year 2013/2014 [29]. Using the average live births between 2013 and 2014 as 912,427 [30] and the average number of neonatal deaths in the same period (calculated using the neonatal mortality rate obtained from Kenya National Bureau of Statistics et al. [25]) as 20,073; and given that the model estimated that the probability of neonatal mortality reduced by 20.0% as a result of the policy (effectiveness measure from the policy in Table 2*, FE (without first born (column 8))*), the estimates suggest that the FMP translated to on average 4,015 fewer neonatal deaths in 2013/2014. Consequently, using Kenya’s Value of Statistical Life (VSL) of 231,000 [31], the final results show the benefits of USD 927 million from the FMP. These benefits, by far, surpass the actual cost of the FMP. Therefore, the FM cost-to-benefit ratio is 21.22 (from USD 43.7 million to USD 927 million), considering the value of a life saved (the probability of reduction of neonatal mortality as a result of the FMP) (Table 4).

**Table 4:** Per mother and child cost savings calculation

Indicators	Denotation and formula	Amount/ Number
Live births 2013	a	870,599
Live births 2014	b	954,254
Number of live births in 2013–2014 (calculated)	c = (a + b)	1,824,853
Average birth of 2013–2014	d = c/2	912,427
Neonatal Mortality Rate from Kenya National Bureau of Statistics et al. [25]	e	22
Per live births	f	1,000
Average deaths per year (2013–2014)	g = (d × e)/f	20,073
Estimated impact of the policy on Neonatal Death (from Table 2, FE (without firstborn))	h	− 0.200
Number of deaths avoided because of free policy	i = (− g × h)	4,015
Value of statistical life from Viscusi and Masterman [31]	j	231,000
Amount spent on the policy in the financial year 2013/2014 from Mulaki and Muchiri [29]	k	USD 43,700,000
Annual benefit of the policy	l = I × j	USD 927,390,295
Cost-to-benefit ratio	m = l/k	21.22

## Discussion

This study evaluates the causal impact of the FMP in Kenya on early neonatal and neonatal mortality and intermediate outcomes (delivery through CS, skilled delivery, and LBW). We achieve this by using robust programme evaluation approaches of DID [32]. Our study reveals a pronounced (significant) impact of the FMP on reducing both early neonatal and neonatal mortality. These findings could potentially be explained by a significant increase in the use of skilled delivery. While there is a dearth of literature that addresses the effects of the FMP on neonatal mortality [33], the results of this study do not support the findings from earlier studies in Kenya that have linked the FMP to increased neonatal mortality [10–12]. One study that compared the causes of neonatal mortality using a quasi-experimental design before and after the FMP, using facility data in 77 health facilities in 14 counties, showed that neonatal deaths increased from 5,442 to 6,981 [11]. Another study by the same authors using the same data, but that applied a time series analysis of the utilisation of delivery services, neonatal and maternal mortality (two years before and after time series analysis), showed a non-significant reduction in neonatal mortality rates from 23.3 to 22.9 per 1,000 live births (p = 0.14) after the implementation of the FMP [12]. However, their analysis looked at the reduction within the hospitals and did not account for the mortality happening outside the hospitals or may have been unable to catch the whole country's effects of the policy. Thus, this study's results fill in the gap. Besides, given the use of comparable births before and after the policy in our study, having removed heterogenous mother effects, the findings reveal that the probability of early neonatal deaths and neonatal deaths have reduced by 20.5% and 20.2% due to the FMP.

The past decade has seen a consistent reduction of under-five mortality in Kenya due to improved nutrition, wealth, maternal literacy but not user fees related [34]. Therefore, given the design of our study, this finding provides improved estimates compared to other studies towards estimating the causal effect of the FMP and fills the literature gap on the cause and effect of free maternity policies on neonatal and early neonatal mortality.

The other finding is that while there has been an increase in the probability of births through CS by 2.1%, it is not attributable to the policy. This confirms the findings by Lang'at et al. [13], who, in their interrupted time series analysis (2 years before—2 years after the policy) using maternal health indicators reported monthly and collected in three counties in Kenya, showed that there was no significant change in the CS rates after the policy. The non-significant increase could be explained by the fact that only a few facilities (level 4,5, and 6) act as referral facilities to conduct CS as per the *Kenya delivery structure* [35]. Also, CS is only conducted by a medical officer, an obstetrician and a gynaecologist, or until recently, a clinical officer in reproductive health, primarily stationed in the referral facilities [13]. However, in Senegal, the CS significantly increased from 4.2 to 5.6% one year after the abolition of CS fees [36].

Also, during a similar evaluation period, there was a significant increase in the probability of skilled delivery. One possible explanation is that an increase followed the FMP in the facility-based delivery after the policy, which has also been shown in other FMPs [37, 38]. Many women not accessing maternal care before the FMP could consequently be accessing it as a result, as other studies have shown increased utilisation after the FM policies [39–41]. Calhoun et al. [42] showed that because of the FMP in Kenya, poor women were more likely to deliver in healthcare facilities due to the policy and availability of SBAs. Additionally, mothers are more likely to deliver in public facilities (under which the FMP was implemented) than private hospitals because of the policy [42], and it could be attributed to the removal of cost barriers [43]. By removing the cost barriers, the women paying for delivery before the FMP can now use the money to improve their health and focus on having a better pregnancy for the baby. After the introduction of the FMP in 2013, there was a level increase in skilled delivery followed by a trend decrease in CS public facilities [14]. Also, Maina and Kirigia [44] disclosed an increase in public sector deliveries (which are done through skilled delivery) based on facility surveys from chosen counties. However, Tomedi et al. [45], whose work was based on data from 29 rural facilities in the rural HCs in Machakos County seven months after introducing the new policy, did not find an increase in births in public facilities. The difference could be on the limitation of the data type.

Given the robust strategy we have used and the nationally representative data, the findings affirm the increase in the probability of skilled delivery following the FMP. However, other literature has shown that the increase in skilled deliveries without a subsequent effort to address the health system challenge/ gaps (shortage of HCWs, increased workload, shortage of drugs, and delayed reimbursements) may be contributing to neonatal mortality [11, 46, 47], without which the impact of the policy on the mortality (in this study) may have been higher.

Another interesting finding is that the FMP translated to on average 4,015 fewer neonatal deaths in 2013/2014, with a cost-to-benefit ratio of 21.22, showing that the FMP is associated with an incredible return to the country. The findings show that the net benefits are far higher than the costs, indicating that further investment into the FMP could potentially avert even more neonatal deaths.

This study has some limitations. First, due to the data availability, we could not control for some time-varying effects, which have been discussed in the theory of change and data preparation section. However, under the current study design, the policy measure is likely to be exogenous to individual characteristics of women and their families. Secondly, there is a limitation of potential underestimation of the policy effect (due to more deaths being reported now). The clinical distinction between a SB and an early neonatal death can be challenging, especially for home births attended by non-skilled providers. Therefore, it was more likely that the early neonatal death would be considered a stillbirth. Such a misclassification implies the presence of an upward bias in the estimated effect of the FMP, which means that the current estimate is a lower bound (conservative estimated) of the true policy effect. Finally, our study does not allow us to account for sample attrition due to maternal mortality, i.e. we do not capture the outcomes of women who died before the interview. Those women who died before the interview would be the ones with the poorest health endowment and, as a result, a higher likelihood of poor birth outcomes. Hence, the policy would be most effective in improving their birth outcomes compared to mothers with better health endowments. Henceforth, like with the previous limitation, this reconfirms that our estimate represents a lower bound of the true effect and, therefore, can be used as a conservative estimate in the policy considerations.

## Conclusion

Our study has provided a methodological approach to evaluating the FMP's impacts as captured by early neonatal and neonatal deaths and intermediate outcomes. This analysis has moved closer to the causal estimate of the FMP effects. It has been shown that the FMP reduces early neonatal and neonatal mortality. Besides, the FMP is associated with an incredible return to the country. The net benefits are far higher than the costs, which implies that further investment into the FMP could potentially avert even more neonatal deaths. Our findings imply that the policymakers need to look at ways of further expanding and sustainably funding the FMP for even better outcomes.

### Supplementary Information

Below is the link to the electronic supplementary material.

Supplementary file1 (DOCX 55 KB)

## Data Availability

The datasets used in this study are publicly available from the MEASURE DHS programme at ICF International (http://dhsprogram.com/).

## Footnotes

1 Even by including these variables in the regression, we see no change in the outcome of interest, hence, their exclusion in all other regression and they do not appear in the FE specification because they are time invariant.
