# Supplementary material for: Does a free maternity policy in Kenya work? Impact and cost–benefit consideration based on demographic health survey data
Source: Eur J Health Econ. 2023 Feb 13;25(1):77–89. doi: 10.1007/s10198-023-01575-w (PMC10799835; doi:10.1007/s10198-023-01575-w)
Supplement: Supplementary file 1 — Supplementary file1 (DOCX 55 KB) [file 10198_2023_1575_MOESM1_ESM.docx]

**Supplementary materials**

**Appendix 1: Literature review that underpins the theory of change**

The theory of change underpinning this study was based on a review of the literature on the different characteristics that elucidate the expected impact of the FMP on maternal and infant outcomes. For instance, the utilisation of ANC services stimulates familiarity of the mothers with the health care system and allows the healthcare workers (HCWs) to promote skilled delivery services [1], whilst skilled attendance and urine analysis provided during pregnancy play a critical role in determining the outcome of a delivery and neonatal mortality [2]. Other ANC services such as checking for blood pressure and intake of iron-folic acid supplement decrease the risk of preterm birth, LBW, anaemia, and neonatal mortality [3]. Also, a higher number of the ANC visits increases the chances of early identification of complications leading to better delivery outcomes whilst tetanus injections prevent mothers from acquiring tetanus, and lack of it is associated with neonatal mortality [4].

Maternal characteristics have been shown to have an association with attending skilled delivery and potential for affecting neonatal mortality. For example, infants born to younger (under age 18) or older (over age 34) mothers had a higher probability of dying in early childhood; and it was twice as likely for a baby to die if the mother had a birth interval between the children of less than two years compared to three or more years [5]. There were six times higher deaths in the neonatal period among neonates who were multiple births than single births [6], and ethnicity was the second most important determinant after breastfeeding of the socio-demographic characteristics of infant mortality in Kenya [7]. The authors argued that there was a significant socio-economic inequality between the mothers based on their ethnicity attributable to numerous socio-economic advantages such as high urbanisation of their regions, greater wealth, and a low proportion of non-educated women among some groups [7]. Also, mothers' level of education is a contributor to seeking delivery services, child survival and, therefore, associated with better health-seeking behaviour [8]. For instance, more educated women are likely to marry and get to motherhood later, get fewer children, utilise the ANC services and immunise children [9].

On household wealth, children from wealthier households are at a lower neonatal and post-neonatal mortality risk compared to those from poorer backgrounds [10]. Similarly, region of residence is a significant determinant of both neonatal and post-neonatal deaths in Kenya, potentially due to differences in the socio-economic and ecological environmental conditions [10]. Area or place of residence – categorised as either rural or urban – is a crucial socio-economic determinant of infant mortality in Kenya; and the availability of health services in urban areas could enhance the survival of the child [10]. Besides, place of delivery and skilled assistance plays a significant role in maternal and neonatal birth outcomes in that there could be a 29% reduction in neonatal mortality if mothers deliver in health facilities [11]. Infants delivered at home under the care of traditional birth attendants are six times more likely to die than infants born in a hospital [12]. Also, the potential for death among neonates born through CS is significantly high [13], although this estimate is not causal, given that doctors recommend CS to high-risk pregnancies, which is related to higher mortality risks to the newborns, and not because of the procedure.

Besides, the gender of the baby, birth size, APGAR (**A**ppearance (skin colour); **P**ulse (heart rate); **G**rimace response (reflexes); **A**ctivity (muscle tone); **R**espiration (breathing rate and effort)) score of the baby, and early initiation of breastfeeding have been identified as critical determinants of maternal and neonatal outcomes. The mortality rates among male children in the early neonatal period are significantly higher than among female children [12] as males are more likely to die in their first year of life than females [6]. Neonates who are born with LBW, often defined as being less than 2500 grams [14] are twice as likely to die in their first month of life than those whose weight is categorised as average or larger [5]. Additionally, the risk of neonatal death is six times higher among neonates born with low APGAR scores as compared to those with a normal APGAR score and that neonates who had both LBW and the low APGAR score had 28 times higher risk of neonatal death compared with those with normal APGAR scores [12]. On the other hand, failure to initiate early breastfeeding increases the risk of neonatal death [15, 16]. For instance, neonates who were not initiated on early breastfeeding had 20 times more risks of death than those who were breastfed early [12], 16% of neonatal mortality was preventable if all infants were breastfed from the first day, and 22% preventable deaths if breastfeeding started within the first hour [17].

Also, variables outside the health services, such as the availability of drinking water and toilet facilities in homes, significantly affect maternal and neonatal outcomes. For instance, children from households without a water supply and a toilet facility have a significantly higher risk of both neonatal and post-neonatal deaths than children who had both in their homes [10].

**Appendix 2: Data preparation**

***Outcome variables:*** The two main outcome variables are **a)** early neonatal mortality (deaths to live births within the first seven days of life); and **b)** neonatal mortality (deaths at ages 0 to 30 days, including deaths reported at age zero months). The two were calculated using the reported age at death. I also explored other intermediate outcomes under ANC care, delivery care, neonatal factors and mother and child postnatal care as follows:

*ANC care:* *Attended to by skilled assistants in pregnancy* was recoded from question 409 of 2014 KDHS to reflect skilled service provision by either a doctor, nurse, or a midwife [5]. *The variable was binary: 0 – "No", and 1 – "Yes." Weight check, height check, blood and urine sample taken in pregnancy* were recorded from question 413 of the questionnaire of 2014 KDHS that asked if any of the above was conducted at least once in the last birth and were binary: 0 – “No”, and 1 – “Yes” (ibid, 2014, p455*). Iron tablets taken in pregnancy* was curated as binary: 0 – “No”, and 1 – “Yes” from the question 421 of the questionnaire that asked if, during the last birth, the mother was given or bought iron tablets/syrup (ibid, 2014, p421). The response ‘do not know’ was treated as missing. Variable *took intestinal parasite drugs* curated as binary: 0 – “No”, and 1 – “Yes” from the *question 423 of the questionnaire* that asked if the mother took intestinal parasite drugs in the last pregnancy (ibid, 2014, p456). The response ‘do not know’ was treated as missing. *Malaria prophylaxis in pregnancy* was recoded as 0 – “No”, and 1 – “Yes” to reflect whether the respondent received malaria prophylaxis in pregnancy or not. It was based on responses to questions 425-429 on the questionnaire. The *timing of the first ANC* and the number of ANC visits during pregnancy were curated following the MoH recommendations [18]. For instance, that first visit was recoded as 1 if it happened '16 weeks or less’, and 0 – ‘above 16 weeks,’ and the *number of ANC visits* were recoded as 1 – ‘4 and above ANC visits during pregnancy’, and 0 – ‘less than four’ (ibid, 2012).

*Delivery care: Delivery through the CS* which was obtained from question d *‘was (NAME) delivered by caesarean, that is, did they cut your belly open to take the baby out?’* [5]. The question targeted all births that happened in the last five years before the interview. The outcome variables were binary: 0 – “No”, and 1 – “Yes.” Equally, *assistance by skilled assistants at delivery* was recoded to reflect skilled service provision at birth by either a doctor, nurse, or a midwife; the variable variables were binary: 0 – “No”, and 1 – “Yes” and birth in public hospital birth was considered to reflect the operationalisation and implementation of FM policy deliveries in all public facilities (including hospitals) and not private for-profit or not for profit facilities [19].

*Neonatal factors: Low birth weight (birth size)* was recorded based on Nakimuli et al.’s [14] definition of LBW being that less than 2500 grams; hence were categorised as either; 0 – ‘No’ and 1 – ‘Yes.’ *initiation of breastfeeding* was categorised as 1 – ‘immediately’; 0 – ‘After 1 hr or later’ based on Debes et al.’s [15] definition of early breastfeeding.

*Postnatal care:* both variables of *the mother being checked by a health professional after delivery* and *babies with a postnatal check within two months* were recoded as binary: 0 – “No”, and 1 – “Yes” based on the interview responses.

***Explanatory variables:*** They were categorised as either demographic characteristics of the mother, socio-economic characteristics, and neonatal characteristics. Data were categorised and prepared as follows: *Age of the mother at birth of the baby* was constructed using the variable century month code (CMC) which is described as the difference between 1,900 and year of an event, multiplied by twelve and then adding the month of the event [20]. The value was calculated by taking the CMC at the birth of the child minus CMC at the birth of the mother then divided by 12 as no question explicitly asked about the year. It was categorised as 1 – ‘Less than 20’; 2 – ’20-34’; and 3 – ‘35 years and above’ and the breakpoints were selected because they captured three reproduction trajectories of adolescence, young adults, and older adults as also been used by Mustafa and Odimegwu [7]. *Age of the mother at first birth* was categorised as a binary variable, either 1 – ’18 years and below’, and 0 – ’19 years and above’ to capture the effect of teenage pregnancy on neonatal mortality. *Preceding birth intervals* was categorised binary as either 1 – ‘Less than two years’, and 0 – ‘2 years and above’ constructed from the preceding birth interval (months) variable. *Parity,* which Kozuki et al. [21] define as the number of times a woman has given birth to a live or dead foetus of a gestation age of 24 or more weeks, was constructed as 0 – ‘parity 1, 2, and 3’; and 1 – ‘parity 4 and above’ to capture parities as either low multiparity or grand multipara as suggested by Bai et al. [22]. I used the variable *bord* (birth order) to curate it as noted in the DHS 7 guidelines [20]. *Multiple pregnanc*ies were categorised as binary, either 0 – “No” or 1 – “Yes” whether the mother had twins. *Wealth index (quintile)* was presented as had been collected during the survey 1 – Poorest; 2 – Poorer; 3 – Middle; 4 – Richer; and 5 – Richest. *The religion of the mother* was constructed as a vector of dummies for each of the categories of the variables under religion. The categories of the variables were 1 – Roman catholic; 2 – Other Christian; 3 – Muslim; 4 – Other (those with no religion were combined in the category ‘other’). Mother’s occupation was constructed as either 0 – ‘not working’ or 1 – ‘working’. Given Kenya’s great diversity of culture and people; and with more than 42 main ethnolinguistic minorities (tribes) [23, 24], *the ethnicity of the mother* was classified into seven broader categories following Mustafa and Odimegwu’s [7] classification. The categories were 1 – ‘Kalenjin’; 2 – ‘Kamba’; 3 – ‘Kikuyu’; 4 – ‘Luhya’; 5 – ‘Luo’; 6 – ‘Somali’ and last category 7 were all the rest of the mothers whose ethnic categorisation had less than 50 observations. *Highest level of education of the mother* was constructed using a vector dummy of each category, with the categories being as 1 – ‘Primary’; 2 – ‘secondary’; and 3 – ‘Higher’ and 4 – ‘No education.’ *Gender of the baby* was categorised as 0 – ‘female’; and 1 – ‘male.’

Two economic status of the mother were categorised as follows: *Available toilet facilities* were categorised as 0 – ‘Not available’;1 – ‘Yes Available’. Responses about toilet facilities of ‘no facility’, ‘bush/field’, ‘other’, ‘not a dejure resident’ of variable v116 were classified as not available. The question on the *source of drinking water* could not be classified into either ‘available’ or ‘not available’ since each of the respondents indicated some source of drinking water. Instead, I used classifications as described by Ikamari [10] where they were classified as 1 – ‘Piped’; 2 – ‘Well’; or 3 – ‘Other sources.’ The response ‘other sources’ was treated as either spring, rain, open, tanker, carts, bottled, other or not a dejure residence from the variable v113 (which was based on the question to the interviewees about their source of water).

**Appendix 3: Summary of variables, their definitions, and descriptive statistics**

Supplementary Table 1: Summary of variables, their definitions, and descriptive statistics

| **Variable** | **Description** | **Mean** | **SE** |
| --- | --- | --- | --- |
| **Dependent variable** |  |  |  |
| ***Main outcome variables*** |  |  |  |
| Early neonatal mortality | Dichotomous variable indicating if early neonatal mortality, 0 otherwise | 0.0161 | 0.0009 |
| Neonatal mortality | Dichotomous variable indicating if neonatal mortality, 0 otherwise | 0.0234 | 0.0011 |
| ***Intermediate outcome variables (ANC care)*** |  |  |  |
| Attended to by skilled assistants in pregnancy (assisted by doctor /nurse/midwife) | Dichotomous variable indicating if attended to by skilled assistants in pregnancy, 0 otherwise. | 0.6646 | 0.0033 |
| Timing of the first ANC check is less than 16 weeks | Dichotomous variable indicating if the timing of the first ANC check is less than 16 weeks, 0 otherwise | 0.2570 | 0.0030 |
| Four and above ANC visits during pregnancy | Dichotomous variable indicating if four and above ANC visits during pregnancy, 0 otherwise | 0.3860 | 0.0034 |
| Blood pressure measured in pregnancy | Dichotomous variable indicating if blood pressure measured in pregnancy, 0 otherwise | 0.3024 | 0.0032 |
| Urine sample measured in pregnancy | Dichotomous variable indicating if urine sample measured in pregnancy, 0 otherwise | 0.2829 | 0.0031 |
| Blood sample taken in pregnancy | Dichotomous variable indicating if the blood sample was taken in pregnancy, 0 otherwise | 0.3086 | 0.0032 |
| Iron tablets taken in pregnancy | Dichotomous variable indicating if iron tablets were taken in pregnancy, 0 otherwise | 0.2303 | 0.0030 |
| Malaria prophylaxis in pregnancy | Dichotomous variable indicating if Malaria prophylaxis in pregnancy, 0 otherwise | 0.2633 | 0.0030 |
| Weighed in pregnancy | Dichotomous variable indicating if weighed in pregnancy, 0 otherwise | 0.3120 | 0.0032 |
| Height measured in pregnancy | Dichotomous variable indicating if height measured in pregnancy, 0 otherwise | 0.1362 | 0.0024 |
| Told about pregnancy complication | Dichotomous variable indicating if told about pregnancy complication, 0 otherwise | 0.1726 | 0.0026 |
| Took intestinal parasite drugs | Dichotomous variable indicating if took intestinal parasite drugs, 0 otherwise | 0.1046 | 0.0021 |
| ***Intermediate outcome variables (Delivery care)*** |  |  |  |
| Delivery through CS | Dichotomous variable indicating if delivered through CS, 0 otherwise | 0.0675 | 0.0018 |
| Birth in a public hospital | Dichotomous variable indicating if birth in a public hospital, 0 otherwise | 0.4293 | 0.0034 |
| Assistance by skilled assistants at delivery (assisted by doctor /nurse/midwife) | Dichotomous variable indicating if assistance by skilled assistants at delivery, 0 otherwise | 0.5493 | 0.0034 |
| ***Intermediate outcome variables (Neonatal care/ factors)*** |  |  |  |
| Low birth weight | Dichotomous variable indicating if the baby is Low birth weight, 0 otherwise | 0.0212 | 0.0010 |
| Early initiation of breastfeeding | Dichotomous variable indicating if early initiation of breastfeeding, 0 otherwise | 0.2975 | 0.0032 |
| ***Intermediate outcome variables (Postnatal factors)*** |  |  |  |
| Mother checked by a health professional after delivery | Dichotomous variable indicating mother checked by a health professional after delivery, 0 otherwise | 0.1990 | 0.0028 |
| Babies with a postnatal check within 2 months | Dichotomous variable indicating babies with a postnatal check within 2 months, 0 otherwise | 0.2162 | 0.0028 |
| **Independent variables** |  |  |  |
| ***Maternal characteristics (socio-economic, demographic, biological)*** |  |  |  |
| Age of the mother at the birth of the baby | Categorical variable indicating the age of the mother at the birth of the baby |  |  |
| Less than 20 | 1= if the age of the mother at the birth of the baby is less than 20,0 otherwise | 0.1485 | 0.0025 |
| 20-34 | 1= if the age of the mother at the birth of the baby is 20-34,0 otherwise | 0.7247 | 0.0031 |
| 35 years and above | 1= if the age of the mother at the birth of the baby is 35 years and above,0 otherwise | 0.1269 | 0.0023 |
| Age of the mother at first birth if an adolescent | Dichotomous variable indicating the age of the mother at first birth is 18 years and below |  |  |
| 18 years and below | 1= if the age of the mother at first birth is 18 years and below,0 otherwise | 0.4526 | 0.0034 |
| Preceding birth intervals (excluding first-order births) | Dichotomous variable indicating if the preceding birth interval is less than two years |  |  |
| Less than two years | 1= if the preceding birth interval is less than two years,0 otherwise | 0.1915 | 0.0031 |
| Parity | Dichotomous variables indicating grand multipara (parity of 4 and above) |  |  |
| Grand multipara | 1= if parity is four and above, 0 otherwise | 0.1545 | 0.0025 |
| Multiple pregnancy | Dichotomous variable indicating if the multiple pregnancy,0 otherwise | 0.0279 | 0.0011 |
| Ethnicity | Categorical variables indicating the ethnicity of the women |  |  |
| Kalenjin | 1= if ethnicity is Kalenjin, 0 otherwise | 0.1528 | 0.0025 |
| Kamba | 1= if ethnicity is Kamba, 0 otherwise | 0.0782 | 0.0019 |
| Kikuyu | 1= if ethnicity is Kikuyu, 0 otherwise | 0.1130 | 0.0022 |
| Luhya | 1= if ethnicity is Luhya, 0 otherwise | 0.1184 | 0.0022 |
| Luo | 1= if ethnicity is Luo, 0 otherwise | 0.1045 | 0.0021 |
| Somali | 1= if ethnicity is Somali, 0 otherwise | 0.0817 | 0.0019 |
| Other | 1= if ethnicity is Other, 0 otherwise | 0.3515 | 0.0033 |
| Occupation | Dichotomous variable indicating if working,0 otherwise | 0.3031 | 0.0032 |
| Wealth index (quintile) | Categorical variables indicating Wealth index of the women |  |  |
| Poorest | 1= if wealth index is poorest, 0 otherwise | 0.3423 | 0.0033 |
| Poorer | 1= if wealth index is poor, 0 otherwise | 0.2074 | 0.0028 |
| Middle | 1= if wealth index is middle, 0 otherwise | 0.1667 | 0.0026 |
| Richer | 1= if wealth index is richer, 0 otherwise | 0.1493 | 0.0025 |
| Richest | 1= if wealth index is richest, 0 otherwise | 0.1343 | 0.0024 |
| Religion | Categorical variables indicating religion of the women |  |  |
| Roman Catholic | 1= if religion is Catholic, 0 otherwise | 0.1835 | 0.0027 |
| Other Christian | 1= if religion is Christian, 0 otherwise | 0.6180 | 0.0034 |
| Muslim | 1= if religion is Muslim, 0 otherwise | 0.1681 | 0.0026 |
| Other | 1= if religion is other, 0 otherwise | 0.0286 | 0.0012 |
| Type of place of residence | Dichotomous variable indicating if the type of residence of the mother |  |  |
| Urban | 1= if the type of residence is urban, 0 otherwise | 0.3260 | 0.0032 |
| Region of residence | Categorical variables indicating the region of residence |  |  |
| Coast | 1= if Coast, 0 otherwise | 0.1264 | 0.0023 |
| North Eastern | 1= if North Eastern, 0 otherwise | 0.0760 | 0.0018 |
| Eastern | 1= if Eastern, 0 otherwise | 0.1441 | 0.0024 |
| Central | 1= if Central, 0 otherwise | 0.0678 | 0.0017 |
| Rift Valley | 1= if Rift Valley, 0 otherwise | 0.3267 | 0.0032 |
| Western | 1= if Western, 0 otherwise | 0.0942 | 0.0020 |
| Nyanza | 1= if Nyanza, 0 otherwise | 0.1392 | 0.0024 |
| Nairobi | 1= if Nairobi, 0 otherwise | 0.0254 | 0.0011 |
| Highest level of education of the mother | Categorical variables indicating the highest level of education of the women |  |  |
| Primary | 1= if the highest level of education of the mother is primary, 0 otherwise | 0.5272 | 0.0035 |
| Secondary | 1= if the highest level of education of the mother is secondary, 0 otherwise | 0.1909 | 0.0027 |
| Higher | 1= if the highest level of education of the mother is higher, 0 otherwise | 0.0630 | 0.0017 |
| No education | 1= if the highest level of education of the mother is none, 0 otherwise | 0.2189 | 0.0029 |
| ***Neonatal factors*** |  |  |  |
| Gender of the baby | Dichotomous variable indicating if the male gender of the baby |  |  |
| Male | 1= if the gender of the baby is male, 0 otherwise | 0.5073 | 0.0035 |
| ***Economic status*** |  |  |  |
| Source of drinking water | Categorical variables indicating the source of drinking water |  |  |
| Piped | 1= if the source of drinking water is piped, 0 otherwise | 0.3063 | 0.0032 |
| Well | 1= if the source of drinking water is well, 0 otherwise | 0.2497 | 0.0030 |
| Other | 1= if the source of drinking water is other, 0 otherwise | 0.4204 | 0.0034 |
| Toilet facilities available | Dichotomous variable indicating the availability of toilet | 0.7416 | 0.0030 |
| *Note: The description of the data is when all outcome variables have equal observations – (n= 20,927).*  *The first column shows the variable, and the second describes the categories.* | | | |

**Appendix 4: Exploration of the model**

To further explore the mechanism for the change, we explored the impact of the policy using five variations of the basic specification of the FE model (without first born in the sample):

*Panel A:* is a mother fixed effect model exploring the use of skilled delivery as an additional control rather than an outcome informed by mixed findings in literature. For instance, in Kenya, skilled delivery has reduced both neonatal and under-five mortality [25], while an analysis of pooled DHS data from nine countries in Asia, Africa, and Latina America/Caribbean showed that there was no association of skilled delivery with a reduction of neonatal mortality particularly in Africa and Asia [26]. The probability of early neonatal death significantly reduces by 18.7% while that of neonatal death reduces by 18.4% due to the policy *(Supplementary Table 2)* compared to 20.6% and 20.0% respectively *(Table 2 main paper, FE (without first born (column 8)))* if skilled delivery is not a control. However, the probability of delivery through CS, though not significant, decreased by 2.9% *(Supplementary Table 2)(Column 3)* as compared to 2.1% *(Table 3 main paper, FE (without first born (column 4))).* This suggests that the skilled delivery does contribute to the reduction in neonatal mortality, but there are other mechanisms for the policy effects, which need to be explored in the future.

*Panel B:* is a mother fixed effect model exploring the use of birth in a public hospital as an additional control because the FMP as implemented in 2013 was operationalised to cater for deliveries in all public facilities (including hospitals) and not private for-profit or not for profit facilities [19]. As can be seen from the *(Supplementary Table 2) (Columns 1, 2 and 3),* there is no significant difference in the estimated effects once the delivery at the public facility is controlled for. Therefore, the public facility (hospital) cannot be considered responsible for the estimated effect of the policy on early neonatal and neonatal mortality.

*Panel C:* is a mother fixed effect model – which in addition to skilled delivery and birth in a public hospital being utilised as a control – is utilising an interaction term of being born after the policy and delivery in a public hospital*.* The interaction term captures the DiD effect which is a valid estimate of the FMP effect if there is 'baseline uniformity across time’ for births in a public hospital [27]. The other time-varying processes are supposed to have a similar impact both before and after implementation of the FMP and the estimate of interaction would capture a variation in the implementation. we expect that there will be no effects of mortality outside of the public hospital. There are no differential effects on the birth in a public facility (hospital) on early neonatal death, neonatal death, and delivery through CS because of the policy *(Supplementary Table 2) (Columns 1, 2 and 3)*. The coefficient of interaction term shows that a reduction in the probability of early neonatal death by 0.5%, an increase in the probability of neonatal death by 0.5%, and a reduction in the probability of delivery through CS by 1.5% closes the policy effect gaps that exist among mothers that gave birth in a public facility and those who did not before the policy was implemented.

*Panel D:* is a mother fixed effect exploring the use of delivery through CS as additional control rather than an outcome and was informed by Signore and Klebanoff’s [13] postulation that potential for death among neonates born through CS is significantly high. The probability of birth being early neonatal mortality significantly reduces by 20.7% while that of neonatal mortality reduces by 20.1% *(Supplementary Table 2)(column 1 and 2)* after the implementation of the policy compared to 20.6% and 20.0% respectively *(Table 2 main paper, FE (without first born (columns 4 and 8)))* if delivery through CS is not a control (nearly similar to if public facility (hospital)).

*Supplementary Table 2: Estimates of the exploration of the impact of the FMP on early neonatal and neonatal mortality, and delivery through CS using mother fixed effects (without first born in the sample)*

|  | **Early neonatal death** | **Neonatal death** | **Delivery through CS** |
| --- | --- | --- | --- |
|  | *(1)* | *(2)* | *(3)* |
| **PANEL A** | *n=1,450* | *n=1,450* | *n=1,444* |
| Born after policy | -0.187** | -0.184** | -0.029 |
|  | (0.077) | (0.078) | (0.030) |
| Skilled delivery | 0.006 | 0.010 | 0.038** |
|  | (0.012) | (0.019) | (0.016) |
| Cons | 0.214** | 0.192** | 0.029 |
|  | (0.086) | (0.093) | (0.044) |
| **PANEL B** | *n=1,467* | *n=1,467* | *n=1,461* |
| Born after policy | -0.205** | -0.202** | -0.028 |
|  | (0.081) | (0.082) | (0.030) |
| Birth in a public facility (hospital) | -0.002 | 0.010 | 0.050*** |
|  | (0.013) | (0.019) | (0.017) |
| Cons | 0.247*** | 0.225** | 0.026 |
|  | (0.091) | (0.097) | (0.043) |
| **PANEL C** | *n=1,450* | *n=1,450* | *n=1,444* |
| Born after policy | -0.185** | -0.188** | -0.024 |
|  | (0.077) | (0.079) | (0.033) |
| Birth in a public facility (hospital) | 0.004 | 0.028 | 0.064 |
|  | (0.017) | (0.030) | (0.045) |
| Skilled delivery | 0.005 | -0.015 | -0.010 |
|  | (0.010) | (0.026) | (0.040) |
| Birth in a public facility (hospital)* born after policy (DiD estimator) | -0.005 | 0.005 | -0.015 |
|  | (0.025) | (0.029) | (0.026) |
| Cons | 0.212** | 0.196** | 0.027 |
|  | (0.087) | (0.094) | (0.047) |
| **PANEL D** | *n=1,460* | *n=1,460* |  |
| Born after policy | -0.207** | -0.201** |  |
|  | (0.081) | (0.082) |  |
| Delivery through CS | -0.033 | -0.037 |  |
|  | (0.088) | (0.087) |  |
| Cons | 0.248*** | 0.228** |  |
|  | (0.091) | (0.097) |  |
| ***Panel A:*** Mother FE, with skilled delivery as an additional control to the model  ***Panel B:*** Mother FE, with birth in a public facility as an additional control to the model  ***Panel C:*** Mother FE, with skilled delivery, birth in a public facility (hospital) as controls and interaction of birth in a public facility (hospital) and born after the policy  ***Panel D:*** Mother FE, with delivery through CS as an additional control to the model  The specifications are the same as *Table 2, main paper* fixed-effects models (plus all additional controls) | | | |

**Appendix 5: Estimates impact of the FM policy on placebo time effect**

*Supplementary Table 3: Estimates impact of the FM policy on placebo time effect*

|  | **Placebo 1** | **Placebo 2** | **Placebo 3** | **Observation (n)** |
| --- | --- | --- | --- | --- |
| Early neonatal mortality | -0.012 | 0.027 | 0.194 | 922 |
|  | (0.042) | (0.036) | (0.243) |  |
| Neonatal mortality | 0.031 | 0.006 | 0.177 | 922 |
|  | (0.037) | (0.037) | (0.241) |  |
| Delivery through CS | -0.013 | -0.024 | -0.102 | 917 |
|  | (0.020) | (0.025) | (0.093) |  |
| Skilled delivery | -0.072 | -0.209* | 0.051 | 907 |
|  | (0.064) | (0.110) | (0.157) |  |
| Birth in a public facility (hospital) | -0.099* | -0.194* | 0.083 | 923 |
|  | (0.057) | (0.102) | (0.125) |  |
| Low birth weight | -0.031 | -0.003 | -0.006 | 923 |
|  | (0.026) | (0.013) | (0.018) |  |

**References**

1. Chama-Chiliba, C.M. and S.F. Koch, *An assessment of the effect of user fee policy reform on facility-based deliveries in rural Zambia.* BMC Research Notes, 2016. **9**: p. 1-14.

2. Schlembach, D., *Urine analysis in pregnancy.* Therapeutische Umschau, 2006. **63**(9): p. 585-589.

3. Zeng, L., et al., *Impact of micronutrient supplementation during pregnancy on birth weight, duration of gestation, and perinatal mortality in rural western China: double blind cluster randomised controlled trial.* BMJ, 2008. **337**: p. a2001.

4. Arunda, M., A. Emmelin, and B.O. Asamoah, *Effectiveness of antenatal care services in reducing neonatal mortality in Kenya: analysis of national survey data.* Global Health Action, 2017. **10**(1): p. 1328796.

5. Kenya National Bureau of Statistics, et al. *Kenya Demographic and Health Survey 2014*. 2014 [cited 2017 5th May]; Available from: <https://dhsprogram.com/pubs/pdf/fr308/fr308.pdf>.

6. World Health Organisation. *Neonatal and Perinatal Mortality: Country, Regional and Global Estimates*. 2006 [cited 2020 06 May]; Available from: <https://apps.who.int/iris/bitstream/handle/10665/43444/9241563206_eng.pdf;sequence=1>.

7. Mustafa, H.E. and C. Odimegwu, *Socioeconomic determinants of infant mortality in Kenya: analysis of Kenya DHS 2003.* Journal of Humanities and Social Sciences, 2008. **2**(2): p. 1-16.

8. Caldwell, J.C., *Education as a factor in mortality decline an examination of Nigerian data.* Population Studies, 1979. **33**(3): p. 395-413.

9. Hobcraft, J., *Women's education, child welfare and child survival: a review of the evidence.* Health Transition Review, 1993. **3**(2): p. 159-175.

10. Ikamari, L.D., *Regional variation in neonatal and post-neonatal mortality in Kenya.* African Population Studies, 2013. **27**(1): p. 14-24.

11. Tura, G., M. Fantahun, and A. Worku, *The effect of health facility delivery on neonatal mortality: systematic review and meta-analysis.* BMC Pregnancy and Childbirth, 2013. **13**(1): p. 18.

12. Abdullah, A., et al., *Risk factors associated with neonatal deaths: a matched case–control study in Indonesia.* Global Health Action, 2016. **9**(1): p. 30445.

13. Signore, C. and M. Klebanoff, *Neonatal morbidity and mortality after elective cesarean delivery.* Clinics in Perinatology, 2008. **35**(2): p. 361-371.

14. Nakimuli, A., et al., *Still births, neonatal deaths and neonatal near miss cases attributable to severe obstetric complications: a prospective cohort study in two referral hospitals in Uganda.* BMC Pediatrics, 2015. **15**(1): p. 44.

15. Debes, A.K., et al., *Time to initiation of breastfeeding and neonatal mortality and morbidity: a systematic review.* BMC Public Health, 2013. **13**(S3): p. S19.

16. Mullany, L.C., et al., *Breast-feeding patterns, time to initiation, and mortality risk among newborns in southern Nepal.* The Journal of Nutrition, 2008. **138**(3): p. 599-603.

17. Edmond, K.M., et al., *Delayed breastfeeding initiation increases risk of neonatal mortality.* Pediatrics, 2006. **117**(3): p. 380-386.

18. Kenya Ministry of Public Health and Sanitation and Kenya Ministry of Medical Services. *National Guidelines for Quality Obstetrics and Perinatal Care*. 2012 [cited 2020 20 December ]; Available from: <http://guidelines.health.go.ke:8000/media/National_Guidelines_for_Quality_Obstetrics_and_Perinatal_Care.pdf>.

19. Chuma, J. and T. Maina, *Free Maternal Care and Removal of User Fees at Primary-Level Facilities in Kenya*, in *Monitoring the Implementation and Impact—Baseline Report*. 2013, Health Policy Project, Futures Group: Washington, DC.

20. Croft, T.N., et al. *Guide to DHS Statistics*. 2018 [cited 2020 13 November]; Available from: <https://dhsprogram.com/data/Guide-to-DHS-Statistics/>.

21. Kozuki, N., et al., *The associations of parity and maternal age with small-for-gestational-age, preterm, and neonatal and infant mortality: a meta-analysis.* BMC Public Health, 2013. **13**(S3): p. S2.

22. Bai, J., et al., *Parity and pregnancy outcomes.* American Journal of Obstetrics and Gynecology, 2002. **186**(2): p. 274-278.

23. Makoloo, M.O., Y.P. Ghai, and Y.P. Ghai, *Kenya: Minorities, Indigenous Peoples and Ethnic Diversity*. 2005, Nairobi: Minority Rights Group International and CEMIRIDE.

24. Kenya Ministry of East African Community and Regional Development. *About Kenya*. 2019 [cited 2020 15 November]; Available from: <https://meac.go.ke/kenya-peoples-and-cultures/>.

25. Machio, P.M., *Determinants of neonatal and under-five mortality in Kenya: do antenatal and skilled delivery care services matter?* Journal of African Development, 2018. **20**(1): p. 59-67.

26. Singh, K., P. Brodish, and C. Suchindran, *A regional multilevel analysis: can skilled birth attendants uniformly decrease neonatal mortality?* Maternal and Child Health Journal, 2014. **18**(1): p. 242-249.

27. Lee, M.-j. and C. Kang, *Identification for difference in differences with cross-section and panel data.* Economics Letters, 2006. **92**(2): p. 270-276.
